# Investigation of Pharmacogenomic Variants in the CYP (P450) Family in Native American Populations of the Brazilian Amazon

**DOI:** 10.1155/ijog/5804093

**Published:** 2026-06-13

**Authors:** Lilian Marques de Freitas, Juliana Carla Rodrigues Marcellino, Francisco Cezar Aquino de Moraes, Natasha Monte, Ana Caroline Alves da Costa, Rita de Cássia Calderaro Coelho, Kaio Evandro Cardoso Aguiar, André Mauricio Ribeiro dos Santos, João Farias Guerreiro, Ândrea Ribeiro dos Santos, Sidney Emanuel Batista dos Santos, Marianne Rodrigues Fernandes, Ney Pereira Carneiro dos Santos

**Affiliations:** ^1^ Faculty of Medicine, Federal University of Pará, Altamira, Pará, Brazil, ufpa.br; ^2^ Oncology Research Center, Institute of Biological Sciences, Federal University of Pará, High Complexity Oncology Unit, João de Barros Barreto University Hospital, Belém, Pará, Brazil, ufpa.br; ^3^ Laboratory of Molecular Biology Applied to Diagnosis and Pharmacogenomics, Faculty of Pharmaceutical Sciences, University of Sao Paulo, Sao Paulo, Sao Paulo, Brazil, usp.br; ^4^ Laboratory of Human and Medical Genetics, Institute of Biological Sciences, Federal University of Pará, Belém, Pará, Brazil, ufpa.br

**Keywords:** Brazilian Amazon, CYP family, genetic population variability, Native Americans, pharmacogenomics

## Abstract

The cytochrome P450 (CYP) enzyme superfamily is essential for xenobiotic metabolism, detoxification pathways, and the regulation of cellular homeostasis. Genetic variability in CYP genes, together with environmental factors, contributes substantially to interindividual and interethnic differences in drug metabolism and therapeutic response. However, Indigenous populations remain largely underrepresented in pharmacogenomic and population genomics studies, limiting the generalizability of current knowledge. This study is aimed at characterizing genetic variation within CYP genes in Native American populations from the Brazilian Amazon and comparing the observed patterns with those of the five continental populations represented in the 1000 Genomes Project. Whole‐exome sequencing data were generated for 64 Indigenous individuals belonging to 12 distinct Amazonian ethnic groups. Variants were identified and annotated across CYP family genes, and their predicted functional impacts were assessed. A total of 506 genetic variants were detected across 63 *CYP* genes. Variants predicted to have modifying effects constituted the majority (*n* = 300), followed by variants of moderate (*n* = 90), low (*n* = 95), and high impact (*n* = 21). Importantly, 11 variants appear to be novel, with no previous annotation in dbSNP or other publicly available variant databases. Comparative analyses revealed distinct variant distributions relative to global reference populations. To our knowledge, this is the first comprehensive exome‐based characterization of *CYP* gene variation in Indigenous populations from Northern Brazil. These findings provide an important genomic resource for an underrepresented population and offer a foundational framework for future pharmacogenomic research, with potential implications for the development of personalized and culturally appropriate precision medicine strategies in the Brazilian Amazon.

## 1. Introduction

The cytochrome P450 (CYP) enzyme family plays a central role in drug metabolism, detoxification pathways, and cellular homeostasis. These enzymes directly influence drug response, efficacy, safety, bioavailability, and resistance, both in metabolic organs and at primary target sites [1, 2]. In humans, the CYP family of enzymes, particularly those in the *CYP1*, *CYP2*, and *CYP3* families, is responsible for the Phase I metabolism of approximately 70%–80% of clinically used drugs, such as antidepressants and antipsychotics, cardiovascular agents, chemotherapeutics, antiparasitic drugs among others [3–7]. These enzymes, primarily located in the liver and small intestine, transform drugs into more hydrophilic metabolites, facilitating their elimination [7, 8].

The human genome encodes approximately 57 *CYP* genes, organized into 18 families and 43 subfamilies [9, 10]. Genetic polymorphisms and epigenetic modifications in *CYP* genes may provide relevant insights into drug metabolism and may reveal potential molecular targets for precision medicine [2].

Genetic variation in *CYP* enzymes, together with environmental factors, contributes to interethnic and interindividual variability in drug response. Individuals may be categorized as rapid or slow metabolizers, a distinction largely explained by CYP expression patterns, primarily in the liver and intestine [6, 11].

According to the Brazilian government, the Indigenous population includes approximately 1,694,836 individuals—about 0.83% of Brazil′s total population—with a strong concentration in the northern region [12]. Despite increasing interest in the cultural and social context of these groups, their genetic backgrounds remain understudied.

Studies in highly admixed populations demonstrate that individuals with a high proportion of Amerindian ancestry may be more susceptible to drug toxicity and altered therapeutic responses [13–16]. Thus, investigating genetic variants—including single nucleotide polymorphisms (SNPs) and insertion/deletion polymorphisms (INDELs)—in populations with distinct ancestries is essential for identifying clinically relevant variants and discovering novel ones. These findings may support the development of diagnostic tools and personalized therapeutic strategies for Amerindian, admixed, and broader Brazilian populations.

Therefore, this study is aimed at characterizing molecular variants in *CYP* genes in an Amerindian population from the Brazilian Amazon and comparing these data with the five continental populations represented in the 1000 Genomes Project.

## 2. Materials and Methods

### 2.1. Ethics, Consent, and Permissions

This study was approved by both the National Research Ethics Committee (CONEP) and the Research Ethics Committee of the Federal University of Pará (CAE: 20654313.6.0000.5172). All participants and Indigenous community leaders signed an informed consent form; when necessary, translators assisted in explaining the study. Sample collection followed the principles of the Declaration of Helsinki.

### 2.2. Study and Reference Populations

**Figure 1:**
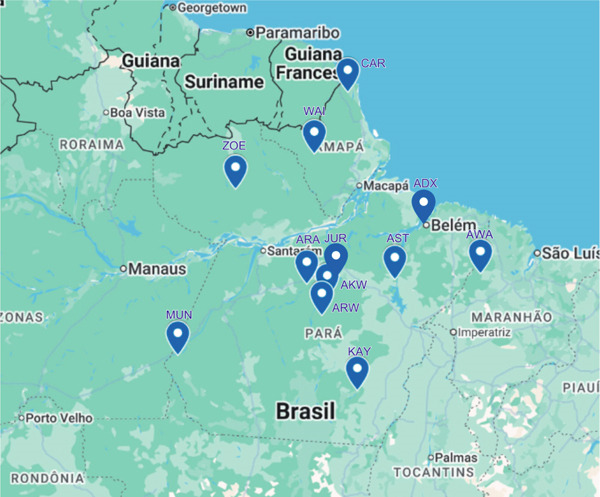
Geographical location of the 12 Indigenous ethnic groups of the Brazilian Amazon evaluated in this study. ARW, Asurini do Xingu; ARA, Arara/Arara do Iriri; AKW, Araweté; AST, Asurini do Tocantins; AWA, Awa‐Guajá; KAY. Kayapó/Xikrin; ZOE, Zo′é; WAI, Wajãpi; CAR, Karipuna; ADX, Phurere; MUN, Munduruku; JUR, Yudjá/Juruna.

The study included 64 individuals from 12 isolated Indigenous ethnic groups of the Brazilian Amazon (Asurini do Xingu, Arara/Arara do Iriri, Araweté, Asurini do Tocantins, Awa‐Guajá, Kayapó/Xikrin, Zo′é, Wajãpi, Karipuna, Phurere, Munduruku, and Yudjá/Juruna) (Figure 1). The samples were collected from adult individuals (between 18 and 50 years old). They were collected as part of two projects developed by the Laboratório de Genética Humana e Médica (LGHM) and approved by the Brazilian National Committee on Research Ethics—CONEP (identified by Nos: 1062/2006 and 123/98). All participants exhibited at least 64% Indigenous ancestry, as determined by a customized ancestry‐informative marker panel. Additional information about the study individuals can be found at [17, 18].

These individuals were grouped into a single cohort referred to as NAM (Native American Population). Reference populations were obtained from the 1000 Genomes Project (available at https://www.1000genomes.org) and gnomAD (available at https://gnomad.broadinstitute.org/ representing five continental groups: Africa (AFR), Europe (EUR), the Americas (AMR), East Asia (EAS), and South Asia (SAS).

### 2.3. Extraction of the DNA and Preparation of the Exome Library

DNA extraction was performed using the phenol–chloroform method (Sambrook 2012). DNA quantity and integrity were assessed via NanoDrop‐8000 spectrophotometry (Thermo Fisher Scientific) and 2% agarose gel electrophoresis.

Exome libraries were prepared using the Nextera Rapid Capture Exome Kit (Illumina) and SureSelect Human All Exon V6 Kit (Agilent). Sequencing was performed on the NextSeq 500 platform with the High‐Output v2 300‐cycle kit.

### 2.4. Bioinformatic Analysis

FASTQ reads were processed using FastQC (v0.11) (https://www.bioinformatics.babraham.ac.uk/projects/fastqc/) for quality control and fastx_tools (v0.13) for filtering (https://hannonlab.cshl.edu/fastx_toolkit/). Sequences were aligned to the GRCh38 reference genome using BWA (v0.7) (https://bio-bwa.sourceforge.net/), indexed with SAMtools (v1.2) (https://sourceforge.net/projects/samtools/), and processed for duplicate removal using Picard Tools (v1.129) (https://broadinstitute.github.io/picard/). Mapping quality recalibration and local realignment were performed using GATK (v3.2) (https://www.broadinstitute.org/gatk/), which was also used for variant calling.

Variant analysis was performed using ViVa software (Viewer of Variants—developed by the Federal University of Rio Grande do Norte, Natal, Rio Grande do Norte, Brazil). Variant annotation was performed using SnpEff v4.3T, Ensembl VEP (Version 99), and ClinVar (v2018‐10). Pathogenicity predictions were generated using SIFT, PolyPhen‐2, LRT, MutationAssessor, MutationTaster, FATHMM, PROVEAN, MetaSVM, M‐CAP, and FATHMM‐MKL.

### 2.5. Variant Selection

Variants were selected based on: (a) minimum depth of 10 reads of quality; (b) impact classification, such as high (disruptive variants likely to cause protein truncation or loss of function), moderate (nondisruptive variants that may alter protein function), modifier (noncoding or regulatory variants with uncertain impact), and low (variants with minimal predicted effect).

### 2.6. Statistical Analyses

Fisher′s exact test was used to compare allele frequencies between NAM and the five continental groups (AFR, AMR, EUR, SAS, and EAS). The Wright′s fixation index (FST) and multidimensional scaling (MDS) metric were used to assess population differentiation. All analyses were performed using Rstudio v.3.5.1, considering as significant a *p* value of < 0.05.

## 3. Results

**Figure 2:**
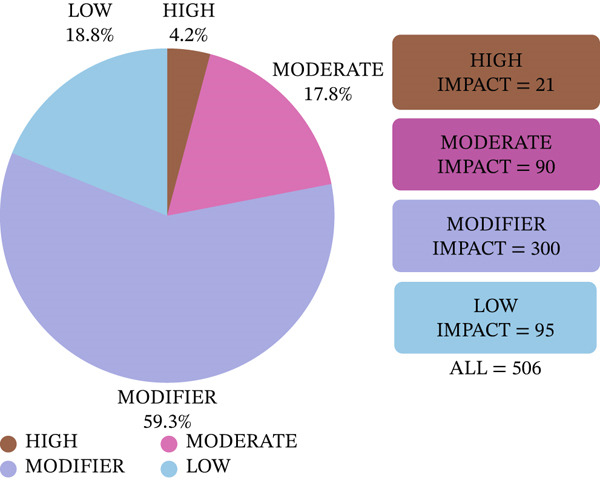
Clinical impact of all genetic variants found.

A total of 645 variants were identified, of which 506 remained after applying selection criteria (Figure 2) (Table S1). Of the previously described variants, 495 were distributed across multiple genes: 20 high impact, 90 moderate, 292 modifier, and 93 low impact. Eleven novel variants were identified, including one high‐impact variant, eight modifier variants, and two low‐impact variants.

**Figure 3:**
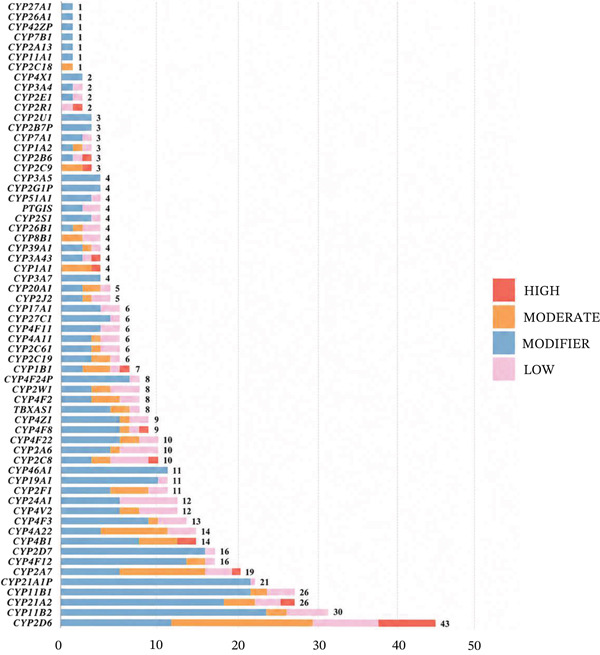
Total distribution of variants by clinical impact of all genes studied.

The gene families with the highest number of variants were *CYP4A* (173), *CYP4F* (75), *CYP21A* (47), and *CYP2D* (40). The genes most enriched for variants included *CYP2D6* (43), *CYP11B2* (30), *CYP21A2* (26), *CYP11B1* (26), and others (Figure 3).

Details about the high‐impact variants, already described in the literature and included in our results, are presented in Table 1.

**Table 1:** Allele frequency in high‐impact variants already described in the 1000 Genomes database.

Gene	SNP	Chromosomal location	Nucleotide change	Protein change	Minor allele frequency					
					NAM	AFR	AMR	EAS	EUR	SAS
*CYP2D6*	rs146271511	chr22:42127458	c.1162C>T	p.(Arg388Cys)	0.0833	> 0.0001	> 0.0001	> 0.0001	—	0.001
*CYP11B2*	rs4539	chr8:142915123	c.518A>G	p.(Lys173Ar)	0.0833	0.188	0.530	0.713	0.516	0.606
*CYP2D6*	rs149012039	chr22:42142513	c.478dupG	p.(Glu160Glyfs∗43)	0.1015	0.489	0.231	0.571	0.297	0.274
*CYP2D6*	rs202102799	chr22:42127556	c.1064A>G	p.(Tyr355Cys)	0.0833	0.0003	0.002	0.0002	> 0.0001	0.0095
*CYP21A2*	rs6464	chr6:32038560	c.138C>A	p.(Pro46=)	0.8438	—	—	—	—	—
*CYP2A7*	rs200892263	chr19:40877242	c.1109T>A	p.(Leu370∗)	0.1667	—	0.006	—	—	—
*CYP1A1*	rs141173079	chr15:74722797	c.301G>A	p.(Asp101Asn)	0.0833	—	—	—	0.001	—
*CYP3A43*	rs61469810	chr7:99836454	c.74delA	p.(Tyr25Leufs∗65)	0.0313	—	—	—	—	—
*CYP1B1*	rs750220338	chr2:38070798	c.1556A>C	p.(Asn519Thr)	0.0259	—	—	—	—	—
*CYP2C9*	rs1799853	chr10:94942290	c.430C>T	p.(Arg144Cys)	0.0172	0.008	0.099	0.001	0.124	0.035
*CYP2B6*	rs140830969	chr19:41012413	c.1080C>T	p.(Asp360=)	0.0833	—	0.004	—	—	—
*CYP2D6*	rs1058172	chr22:42127526	c.1094G>A	p.(Arg365His)	0.0833	—	—	—	—	—
*CYP2D6*	rs1065852	chr22:42130692	c.100C>T	p.(Pro34Ser)	0.0833	0.113	0.148	0.571	0.202	0.165
*CYP2C8*	rs138495387	chr10:95037276	c.1325G>T	p.(Cys493Phe)	0.1	—	—	—	—	—
*CYP2D6*	rs28371726	chr22:42127537	c.1083T>C	p.(His361=)	0.0833	—	—	—	—	—
*CYP2R1*	rs12794714	chr11:14892029	c.177C>T	p.(Asp59=)	0.5469	0.103	0.514	0.368	0.447	0.445
*CYP4B1*	rs150307866	chr1:46810912	c.285T>A	p.(Tyr95∗)	0.0833	—	—	—	0.001	—
*CYP2D6*	rs3892097	chr22:42128945	c.506‐1G>A	p.(?)	0.0288	0.061	0.13	0.002	0.186	0.109
*CYP4B1*	rs3215983	chr1:46815074	c.884_885delAT	p.(Asp295Glyfs∗3)	0.2188	0.039	0.182	0.255	0.13	0.116
*CYP21A2*	rs6469	chr6:32040674	c.1125C>T	p.(Ser375=)	0.1667	—	—	—	—	—

**Figure 4:**
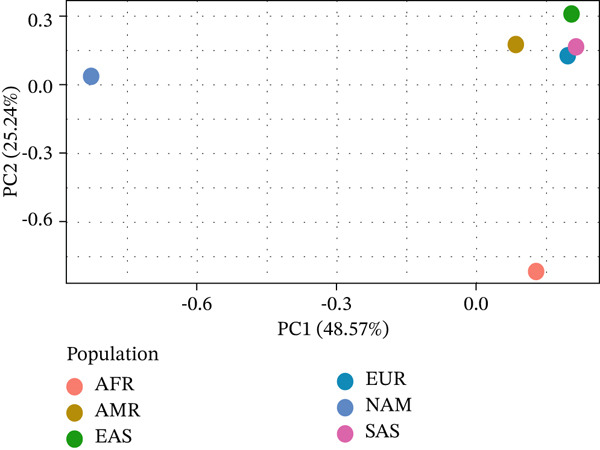
Variants analyzed in the six populations of the present study. AFR, African; AMR, American; EAS, East Asian; EUR, European; NAM, Amazonian Amerindian; SAS, South Asian.

Allele frequency comparisons demonstrated that the NAM population exhibits a distinct genetic profile relative to the continental reference groups, particularly AFR, which also represents an ancestral population with substantial diversity (Figure 4).

Among the 11 novel variants (Table 2), one high‐impact frameshift mutation was identified in *CYP4F8* on Chromosome 19, representing an insertion from G to GC in a coding region. Most newly identified variants were in *CYP2D6* (*n* = 3) and *CYP19A1* (*n* = 2).

**Table 2:** Frequency of nonpreviously described variants in the NAM population.

Gene	Chromosomal location	Nucleotide change	Amino acid change	Impact	Region	Region detailed	NAM
*CYP4F8*	chr19:15619687	c.451dupC	p.(Leu151∗)	High	CDS	FRAME_SHIFT	1
*CYP19A1*	chr15:51242197	c.145+571A>T	p.(?)	Modifier	Intronic	Intron	0.125
*CYP2F1*	chr19:41122203	c.822+71delC	p.(?)	Modifier	Intronic	Intron	> 0.0001
*CYP21A1P*	chr6:32006751	c.979+30A>C	p.(?)	Modifier	Intronic	Intron	0.1667
*CYP2D6*	chr22:52919	c.180+34C>G	p.(?)	Modifier	Intronic	Intron	0.6613
*CYP2D6*	chr22:52912	c.180+41A>C	p.(?)	Modifier	Intronic	Intron	0.6167
*CYP4F11*	chr19:15934183	c.198+28G>C	p.(?)	Modifier	Intronic	Intron	0.0833
*CYP19A1*	chr15:51242191	c.145+576_145+577insC	p.(?)	Modifier	Intronic	Intron	0.1
*CYP2D6*	chr22:52910	c.180+43G>C	p.(?)	Modifier	Intronic	Intron	0.5306
*CYP3A4*	chr7:99778057	c.189A>G	p.(Glu63=)	Low	CDS	SYNONYMOUS_CODING	0.0179
*CYP4F22*	chr19:15549131	c.1271‐7C>T	p.(?)	Low	Other	SPLICE_SITE_REGION+INTRON	0.25

## 4. Discussion

The successful implementation of precision medicine requires a detailed understanding of drug metabolism and phenotypic variability across diverse ancestral groups, particularly those that remain underrepresented in genomic research. Latin American populations exhibit substantial Native American genetic influence; however, Indigenous groups from this region continue to be markedly understudied when compared with European, Asian, or North American cohorts. This lack of representation creates knowledge gaps that impede the application of precision health strategies in these populations [19].

In the present study, we identified 506 genetic variants across 63 CYP genes. Previous exome‐based investigations conducted by our group reported far fewer variants overall. For example, studies involving gastric cancer–related genes [20], the *PCLO* gene [21], COVID‐19 susceptibility pathways [22, 23], and radiotherapy response genes [24], each revealed considerably smaller variant inventories. These comparisons emphasize the substantial complexity and polymorphic diversity inherent to the CYP superfamily, even within populations characterized by relatively reduced global genetic variability.

Given all the variants analyzed, modifying impact constituted the majority (*n* = 300), followed by moderate (*n* = 90), low (*n* = 95), and high‐impact variants (*n* = 21). Notably, 11 variants identified here appear to be novel, with no previous entries in dbSNP or comparable databases. The discovery of previously unreported CYP variants in Amazonian Indigenous groups is consistent with earlier findings from Coelho et al. [24], who likewise reported novel and modifier variants in similar populations, and Aguiar et al. [20], who also observed a predominance of modifier variants, reinforcing the pattern documented in the present study.

The human CYP repertoire comprises 57 functional genes whose allelic architecture is strongly shaped by ancestry, generating distinct metabolic phenotypes such as poor, intermediate, extensive, and ultrarapid metabolizers [9]. *CYP2D6* and *CYP2C19* together contribute to the metabolism of approximately 30% of all approved medications [25], and more than 200 FDA‐labeled drugs contain pharmacogenomic information specifically related to *CYP2D6* catalogued in the ClinPGx database. Here, we have identified 43 *CYP2D6* variants; seven of which are predicted to have high functional impact, underscoring the clinical relevance of this locus. *CYP2D6* variation influences the metabolism of antineoplastic agents, psychotropic and antidepressant medications, neuroleptics, and antiarrhythmics [3, 5, 26, 27]. Given its highly polymorphic nature and marked interethnic variability, characterizing *CYP2D6* diversity in Indigenous populations is crucial for advancing equitable pharmacogenomic implementation [28, 29].

Substantial variation was also observed in *CYP11B1*, *CYP11B2*, and *CYP21A2*, all of which are central to adrenal steroid biosynthesis. *CYP11B1* and *CYP11B2* catalyze the terminal steps in glucocorticoid and mineralocorticoid production, respectively [30]. Variants in *CYP11B2* have been linked to differential responses to antihypertensive therapies such as angiotensin II receptor blockers [31]. *CYP21A2*, which catalyzes key conversions in corticosteroid synthesis, is known to harbor more than 1300 documented variants, and genetic alterations in this gene influence systemic hormone levels and responsiveness to endocrine‐targeted therapeutics [32].

The NAM population also exhibited notable absences of certain high‐frequency global variants. The *CYP2C9* variant rs1799853, a major determinant of warfarin metabolism, was not detected. Another study has emphasized the importance of identifying this variant to optimize warfarin therapy and avoid life‐threatening complications [33]. Similarly, the well‐known *CYP2D6* variant rs3892097 was absent despite the presence of several other high‐impact *CYP2D6* variants detected here. These findings highlight population‐specific patterns that may directly affect therapeutic responses.

Additional noteworthy variants included rs6413419 (*CYP2E1*) and rs3833221 (*CYP2F1*), both present globally but absent in the NAM cohort. *CYP2E1* metabolizes numerous endogenous and exogenous substrates and has polymorphisms associated with alcohol dependence, glioma, motor neuron disease, and polyneuropathy [34]. *CYP2F1*, conversely, participates in the bioactivation of pneumotoxic agents [35]. Their absence may reflect unique metabolic or protective characteristics within Indigenous Amazonian groups.

The *CYP4F* family, which participates in lipid metabolism and regulates gene expression in multiple tissues [36], also merits attention. Prior studies have linked *CYP4F8* variants to methotrexate toxicity in [37] and to gemcitabine response [38]. Thus, the identification of a novel high‐impact frameshift variant in *CYP4F8* is particularly significant. Frameshift events often produce truncated or dysfunctional proteins, but they may also generate neofunctionalization events that become evolutionarily fixed in populations [39–41]. Whether the novel *CYP4F8* variant discovered here alters pharmacologic phenotypes in the NAM group warrants future study.

MDS results corroborate the distinctiveness of the NAM genetic profile relative to continental reference groups. Such differentiation reinforces the need for region‐specific clinical and pharmacogenomic guidelines in the Amazon [14, 16]. Genetic variability across populations is strongly correlated with metabolic phenotypes and drug transport mechanisms [19]. As highlighted in previous studies, African populations exhibit exceptional *CYP450* diversity [42], whereas Asian groups show elevated frequencies of *CYP2C19* poor metabolizers and substantial heterogeneity in drug response [43]. These examples underscore the broader importance of ancestry‐aware pharmacogenomics (PGx).

Despite the promising nature of our findings, some limitations must be acknowledged. Whole exome sequencing (WES) has been widely applied in PGx; however, several technical limitations restrict its ability to comprehensively characterize pharmacogenetic variation. One major limitation is the uneven coverage of clinically relevant PGx genes, particularly those located in complex genomic regions. Genes such as *CYP2D6* may show incomplete or unreliable coverage due to high sequence homology with nearby pseudogenes, such as *CYP2D7*, which can compromise variant detection [44]. In addition, WES primarily targets coding regions, representing only a small fraction of the genome, and therefore fails to systematically capture regulatory variants located in noncoding regions that may influence gene expression and drug response [45].

Another important limitation is the reduced ability of WES to detect structural variation, including copy number variations (CNVs), gene rearrangements, and complete gene deletions that are particularly relevant in pharmacogenes. Structural alterations affecting *CYP2D6*, such as gene duplications or deletions, may therefore remain undetected using standard exome sequencing approaches [46]. Finally, WES based on short‐read sequencing has limited capacity for haplotype phasing, which can hinder accurate diplotype determination and phenotype prediction in PGx analyses.

Additionally, another limitation of this study is the relatively small sample size, which may not capture the full range of CYP diversity present across Amazonian Indigenous groups. The apparent homogeneity of *CYP4F8* in this cohort may reflect low variability, limited sampling power, or the influence of endogamy, an important sociocultural factor in many Indigenous communities. Future studies with expanded cohorts will be critical for elucidating the pharmacogenomic significance of *CYP4F8* and other CYP clusters in this population.

## 5. Conclusions

In summary, we identified several high‐impact and novel variants with potential clinical implications for drug metabolism, thereby contributing valuable insights into the pharmacogenomic landscape of Indigenous Amazonian populations. This work represents the first comprehensive characterization of CYP family variation in exome data from Indigenous groups of Northern Brazil. Although *CYP2D6* has been extensively studied worldwide, gaps remain regarding its clinical and evolutionary significance in Indigenous contexts, particularly in relation to *CYP4F* family members. Our findings offer an essential foundation for future efforts to develop personalized therapeutic strategies and culturally tailored precision medicine initiatives in the Brazilian Amazon.

## Author Contributions

Lilian Marques de Freitas and Juliana Carla Rodrigues Marcellino have contributed equally to this work.

## Funding

This research was funded by the Conselho Nacional de Desenvolvimento Científico e Tecnológico (CNPq) (https://www.cnpq.br/); the Coordenação de Aperfeiçoamento de Pessoal de Nível Superior (CAPES) (https://www.gov.br/capes/pt-br); the Pró‐Reitoria de Pesquisa e Pós‐Graduação da Universidade Federal do Pará (PROPESP) (https://www.propesp.ufpa.br/); and the Fundação Amazônia Paraense de Amparo à Pesquisa (FAPESPA) (https://www.fapespa.pa.gov.br).

## Disclosure

The funders had no role in the design of the study; in the collection, analyses, or interpretation of data; in the writing of the manuscript, or in the decision to publish the results.

## Conflicts of Interest

The authors declare no conflicts of interest.

## Supporting information


**Supporting Information** Additional supporting information can be found online in the Supporting Information section. Table S1: Total number of variants identified through NAM exome sequencing that passed quality control criteria.

## Data Availability

The data obtained from the public domain are available at gnomAD (https://broadinstitute.org/), and the sequencing data of the Amazonian Amerindian populations are available at the EMBL′s European Bioinformatics Institute database (https://www.ebi.ac.uk/), Reference Number PRJEB35045.
